# Unusual Bronchial Foreign Bodies with Localized Bronchiectasis in Five Children

**DOI:** 10.1155/2019/4143120

**Published:** 2019-12-28

**Authors:** Xi-Ling Wu, Lei Wu, Zhi-Min Chen

**Affiliations:** Department of ‘A', The Children's Hospital, Zhejiang University School of Medicine, National Clinical Research Center for Child Health, Hangzhou, China

## Abstract

Obstructive foreign bodies are uncommon causes of bronchiectasis in children, the causal relationship between foreign body aspiration and bronchiectasis remains unclear. We conducted a review of children who were diagnosed with bronchiectasis due to foreign body retention in a university hospital between 2014 and 2019. Five patents were studied (four boys, one girl; age range: 15 months to 13 years old). Computed tomography showed localized cylindrical bronchiectasis in all five patients. After removal of the foreign body by bronchoscopy, the prognoses were good. Patients with localized cylindrical bronchiectasis should be examined to exclude foreign body. As long as foreign body aspiration is diagnosed early and appropriately removed, the possibility of a lobectomy or even mortality is greatly reduced.

## 1. Background

Bronchiectasis is defined as the presence of reversibly or irreversibly widened bronchi due to damage to the bronchial walls. Common causes include postinfectious, aspiration syndromes, defects in host defenses, genetic syndromes, anatomical defects, and external airway compression [1]. In contrast, foreign body (FB) obstruction is an uncommon cause of bronchiectasis [2]. The causal relationship between cases of FB aspiration and bronchiectasis has not yet been established. In this article, we retrospectively analyzed the clinical characteristic of patients diagnosed with bronchiectasis due to FB retention in our hospital between 2014 and 2019. Here, we wished to provide clinicians with useful data on the characteristics of bronchiectasis that result from FB inhalation.

## 2. Objectives and Design

We retrospectively reviewed five patients diagnosed with bronchiectasis due to FB retention from January 2014 to January 2019. We analyzed the following patient demographics: sex, age, basic disease, symptoms, signs, the nature of FB, FB localization, period of retention, computed tomography (CT) results, bronchographic findings and prognosis.

## 3. Results

**Figure 1:**
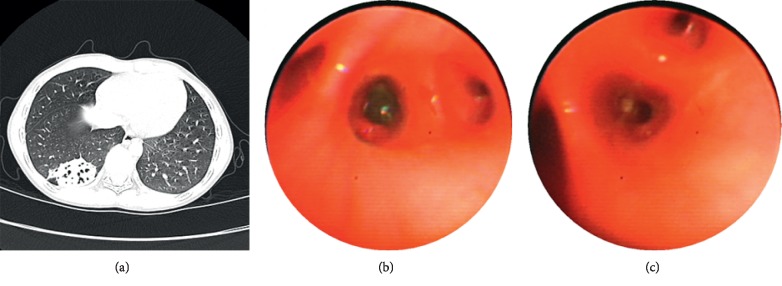
The images of CT and bronchoscopy of case 5: (a) shows bronchiectasis in posterior basal segment of the right lower lobe; (b) shows a spherical foreign body in the posterior basal segment of the right lower lobe (c) shows the picture after removal of foreign body.

Patients characteristics are listed in Table 1. The five patients comprised four boys and one girl with ages ranging from 15 months to 13 years (mean: 6.7 years). The FB identified included a sunflower seed, teeth, two nut, and a tapioca pearl (see Figure 1). The patients presented with recurrent cough, fever, or wheezing; one patient also experienced hemoptysis. Physical examination found wheezing in two cases, wet rales in one case, and decreased breath sounds in one case. One patient had basic disease, and the others were normal. None had histories of typical aspiration. The FB location was the left lower lobe in two patients, and the right lower lobe in three patients. CT showed inflammation, emphysema, and localized bronchiectasis. Bronchographic examination revealed granulation tissue formation in three patients. Repeated X-ray examination showed that bronchiectasis disappeared after 1 or 6 months. In one case, the patient experienced neither fever nor cough for 3 years after removal of the FB and refused repeat X-ray examination.

## 4. Discussion

Aspiration of an FB into the trachea, and subsequently the bronchus, can occur in all age groups and most commonly occurs in infants and little children [3]. Once this occurs, identification of the FB is crucial, and hopefully occurs before bronchiectasis develops; the obstructive increases the risk of chronic infection and subsequent bronchiectasis. The incidence of bronchiectasis due to FB aspiration is reported to be between 1% and 5.6% in the literature [4]. However, approximately 500 patients admitted for FB aspiration in our hospital per year; therefore, the incidence of bronchiectasis in our study was lower than reported.

In our study, the mean age of patients with bronchiectasis due to FB was higher than that of all patients with FB. In addition, all the patients do not have histories of FB aspiration. The FBs were usually organic. CT revealed that bronchiectasis caused by FB was localized cylindrical bronchiectasis, mainly located in the lower lobes.

Aronoff reported in a systematic review that 18 of 989 patients, approximately 2%, with noncystic fibrosis bronchiectasis had FB aspiration [2]. The risk of bronchiectasis depends on the size, shape, localization, and the retention time of the FB [5]. The type of the FB and the time of retention within the tracheobronchial system are the most important factors. It has also been reported that the risk of bronchiectasis increases with retention time from aspiration to diagnosis [6]. Sirmali et al. reported that the earliest case of bronchiectasis developed 25 days after aspiration [5]. However, FB aspiration does not always lead to bronchiectasis, even after 4-5 years. Therefore, the occurrence of bronchiectasis is likely determined by several factors.

Because many patients do not report histories of FB aspiration and do not experience typical aspiration symptoms, this disease is often missed, making diagnosis more challenging. Doctors of patients with impaired mental statuses or swallowing mechanisms should be more vigilant about the possibility of FB inhalation, as was the scenario in one of our cases [7]. CT or bronchoscopic examination should be performed for patients with long-term cough, who are nonresponsive to routine treatment, who experience repeated occurrences of the same symptoms, have recurrent or persistent consolidations in the same location, or have unexplained hemoptysis [8, 9]. Furthermore, obtaining a proper history is crucial. Whenever a choking episode is mentioned in the patient's history, tracheobronchoscopy is indicated without relying on other diagnostic tools [10, 11]. Recently, flexible bronchoscopy is also recommended for children newly diagnosed with bronchiectasis to exclude a foreign body or obstructive lesion [12].

Bronchoscopic removal of a retained FB may be difficult because of the presence of granulation tissue and copious secretions. In one of our case, the granulation tissue was lysed and frozen with liquid nitrogen before removal. In our study, all five patients recovered completely after successful FB removal. After 1–6 months, a repeat CT showed that the bronchiectasis had disappeared. Vergnon reported a case who had bronchiectasis due to pen cap retention for 41 years [13]. The successful removal of the cap leads to significant recovery, avoiding further invasive approach. It has been reported that neglected, inorganic FBs can cause permanent bronchiectasis after several years [14, 15]. These patients sometimes require lobectomy because of recurrent infection after removal of FB. As long as the FB is diagnosed early and removed by bronchoscopy, the need for lobectomy and the risk of mortality can be reduced.

## 5. Conclusion

To reduce the risk of bronchiectasis secondary to FB aspiration, proper histories must be obtained. In addition, CT or bronchoscopic examination should be performed for patients with long-term cough, repeated occurrences of the same symptoms, recurrent or persistent consolidation in the same location in the lungs, or unexplained hemoptysis. Impaired mental statuses or swallowing mechanism in patients should arise suspicion of FB aspiration. Finally, patients with localized cylindrical bronchiectasis should be examined for FB aspiration.

## Figures and Tables

**Table 1:** Clinical data of the five patients.

	Sex/age	Basic disease	Symptoms	Sign	CT	Nature of FB	Site of retention	Period of retention	Bronchographic finding	Prognosis
Case 1	M/15 m'	No	Recurrent cough and wheeze for five months	Wheezing sound in the left lung	Emphysema and bronchiectasis in the left lower lobe	Sunflower seed	Basal segment of the left lower lobe	Five months	Foreign body in the left lower lung with granulation tissue formation	Bronchiectasis basically disappeared in the repeated CT after 5 months
Case 2	M/13 y	No	Cough for seven days and hemoptysis for one day	No positive sign	Inflammation and bronchiectasis in the right lower lobe	Nut	B10 segment of the right lower lobe	Unknown	Foreign body in the right lower lung along with granulation tissue formation	Chest X-ray was normal after two months
Case 3	M/7y	Pachygyria and epilepsy	Fever for eight days and cough for five days	Coarse breath sounds in both lungs	Inflammation and partial bronchiectasis in the left lower lobe	Teeth	Basal segment of the left lower lobe	Unknown	Foreign body in the left lower lung along with granulation tissue formation	Bronchiectasis basically disappeared in the repeated CT after 6 months
Case 4	M/25 m	No	Cough and fever for twelve days	Wet rales in both lungs	Inflammation and bronchiectasis in the right middle and lower lobe	Nut	Right main bronchus	Unknown	Foreign body in the right main bronchus	Bronchiectasis basically disappeared in the repeated CT after 1 month
Case 5	F/10 y	No	Recurrent cough and fever for two months	Right lung breathing sound decreased	Bronchiectasis in the posterior basal segment of the right lower lobe	Tapioca pearl	Basal segment of the right lower lobe	Two months	A spherical foreign body in the right lower lung.	She almost had no fever or cough in recent three years and refused to have X-ray examination
